# Genetic polymorphisms and cervical cancer development: ATM G5557A and p53bp1 C1236G

**DOI:** 10.3892/or.2011.1609

**Published:** 2011-12-22

**Authors:** S. OLIVEIRA, J. RIBEIRO, H. SOUSA, D. PINTO, I. BALDAQUE, R. MEDEIROS

**Affiliations:** 1 Molecular Oncology Group, Portuguese Institute of Oncology of Porto, Rua Dr António Bernardino Almeida, 4200-072 Porto; 2 Molecular Biology Laboratory of Virology Service, Portuguese Institute of Oncology of Porto, Rua Dr António Bernardino Almeida, 4200-072 Porto; 3 Abel Salazar Institute for the Biomedical Sciences (ICBAS), Largo Professor Abel Salazar 2, 4099-003 Porto, Portugal

**Keywords:** ataxia-telangiectasia-mutated protein, p53bp1, genetic polymorphism, DNA damage, human papillomavirus, cervical cancer

## Abstract

Persistent infections by high-risk types of human papillomavirus (HPV) have been established as the etiological agent of cervical cancer. The integration of the HPV genome into the host genome is a crucial step in cervical carcinogenesis, although, correct activation of DNA damage repair pathways will avoid the development of cancer. Recent data indicate that several polymorphisms of key regulators from the DNA damage repair pathway (i.e. 53BP1 and ATM) are associated with cancer development susceptibility. We have developed a hospital-based retrospective study considering 429 cervical specimens from women with different cervical lesions including invasive carcinoma. This study aimed to evaluate the role of the ATM D1853N (5557G>A) and 53bp1 D353E (1236C>G) polymorphisms in the development of cervical cancer, using TaqMan^®^ SNP Genotyping Assays. Statistical analysis revealed that ATM 5557GG homozygous individuals (OR=1.94; p=0.044) are at increased risk of developing LSIL, while for the 53BP1 1236C>G polymorphism no association was found. Nevertheless, we observed a tendency for an increased risk of LSIL in 53BP1 1236C allele carriers (OR=1.63; p=0.069). Logistic regression adjusted for age revealed no significant differences from the non-adjusted analysis. This is the first study to evaluate the role of ATM G5557A and P53BP1 C1236G polymorphisms in cervical cancer susceptibility. This study reveals a possible trend of both polymorphisms for a genetic susceptibility pattern of cervical cancer development. Hence, our results may be of interest for future understanding of the progression of cervical cancer.

## Introduction

Cervical cancer is the third most common cancer in women with over 500,000 new cases and over 275,000 deaths in 2008 according to the World Health Organization (1). Incidence and mortality rates are very different among developed and developing countries, being extremely common in Central and South America, Eastern Africa and South-East Asia (2,3). In Portugal, incidence and mortality rates have been decreasing in the past ten years, and most recently data point to 12.2 and 3.6 per 100,000, respectively (1).

Persistent infection by the oncogenic types of the human papillomavirus (HPV) was established as the main etiological factor for cervical cancer development (4–8). Nevertheless, despite the main role of HPV, epidemiological studies revealed that several co-factors may be implicated in the carcinogenesis model, such as: measures of sexual activity (number of sexual partners, age at first sexual intercourse and sexual behaviour of partners), parity (>3 children), tobacco and alcohol consumption, co-infection with other sexually transmitted agents, as well as immunologic and host genetic factors (7–13).

The integration of the HPV genome in host genome is a crucial event for cell de-regulation towards the development of cervical cancer. Viral DNA integration is only possible with the formation of DNA double-strand breaks (DSBs), which will allow the assimilation of the viral genome linearized into the host genome (14–17). Some studies suggest that the regulation of this process may be of extreme importance in the prevention of cancer development (18).

DSBs are one of the most serious threats to the integrity of the eukaryotic genome and an efficient response to DNA damage is essential for the maintenance of genome integrity, preventing the accumulation of errors (18). Therefore, several proteins from a complex system of different mechanisms are activated to repair the genome (19–21).

When a DSB occurs, p53 binding protein 1 (p53bp1) migrates into its site, promoting the interaction with several proteins involved in DNA repair and/or DNA damage-signalling pathways, including genome guardian protein, p53 (19,22–25). In fact, p53bp1 has been described as the activator of the ataxia-telangiectasia-mutated protein (ATM) response to DNA damage (26,27).

ATM is the key protein in DMA damage response upon DSBs, leading to cell cycle checkpoint activation, DNA repair, gene transcription and in severe cases to apoptosis (28,29). ATM is an important member of the serine/threonine protein kinases family, and several mutations have been associated with cancer development (30–34).

Previous reports have shown that single nucleotide polymorphism (SNP) on both these proteins may be correlated with DSB recognition and cell capacity to repair DNA. An SNP on nucleotide 5557 (exon 9) of ATM, characterized by a G to A transition leads to an amino acid change at position 1853 of the protein (D1853N) (35). This polymorphism was described as affecting an exonic splicing enhancer (ESE), suggesting a possible alteration on the normal splicing of exon 9 of the ATM gene and therefore a production of a less effective protein (36). Previous studies have reported the influence of this polymorphism in the development of several types of cancer (37–42). Furthermore, from the several polymorphisms identified in the coding and promoter regions of the 53BP1 gene (43), a transition C→G at the nucleotide 1236 (exon 9), which leads to an amino acid change at position 353 of the protein (D353E) (44), has previously been associated with the development of breast cancer (43). The aim of this study was to evaluate the role of the ATM D1853N (5557G>A) and p53bp1 D353E (1236C>G) polymorphisms in the development of cervical cancer in Portugal.

## Materials and methods

### Cases and specimens

This study was performed with exfoliated cervical cells collected with cytobrush at the Gynaecological Service of the Portuguese Institute of Oncology of Porto from 429 inpatient women with median age of 45±13.73 years. Samples were used for liquid-based cytology for cytological classification according to the Bethesda classification: 280 normal (45.0±13.23 years), 70 low-grade intraepithelial squamous lesions (44.0±14.82 years), 70 high-grade intraepithelial squamous lesions including *in situ* carcinoma (45±13.7 years), and 9 ICC (47.0±18.85 years) (Table I).

### DNA extraction

DNA was extracted from 200 μl of exfoliated cervical cells using High Pure^®^ Viral Nucleic Acid Roche (Roche Applied Science, Indianapolis, USA) according to the manufacturer’s instructions.

### SNP genotyping

ATM D1853N (5557G>A) and p53bp1 D535E (1236C>G) polymorphisms were genotyped using TaqMan SNP genotyping assays (C__26487857_10 and C___2944794_10, respectively) from Applied Biosystems (Foster City, CA, USA). Reactions were performed on an Applied Biosystems 7300 real-time PCR system (Applied Biosystems) with a 5-μl final volume mixture containing 1X TaqMan genotyping master mix (Applied Biosystems), 900 nM of each primer, 200 nM of probes labeled with either FAM or VIC, and 10 ng of extracted DNA. Thermal cycling conditions were: 10 min at 95°C followed by 40 cycles of 15 sec at 95°C and 1 min at 60°C. Allelic discrimination was performed by measuring end-point fluorescence using ABI PRISM^®^ Sequence Detection System (Version 1.2.3, Applied Biosystems). Results were analyzed by two of the authors, and 10% were randomly selected and re-submitted to genotyping for confirmation. Concordance of genotypes was 100% among replicates.

### Statistical analysis

Genotypes were tested for the Hardy-Weinberg equilibrium (HWE) with the public software available at http://ihg.gsf.de/. Statistical analysis was performed with the computer software SPSS (Statistical Package for Social Sciences version 16.0) for Mac. Chi-square (χ^2^) analysis was used to compare the categorical variables with a 5% significance level. Fisher’s exact test was used for tables containing cells where values are <5 individuals. The odds ratio (OR) and its 95% confidence interval (CI) were calculated as a measure of the association between the genotypes and clinicopathological parameters by including dichotomous covariates for genotypes. Logistic regression analysis was performed by adjusting for age the risk of cervical carcinoma development.

## Results

### 53BP1 C1236G polymorphism

Table II shows the distribution of the 53BP1 1236 G>C genotypes according to cytological classification. The frequency of the 53BP1 1236 GG, GC and CC genotypes were 38.2, 45.5 and 16.3%, respectively. Statistical analysis revealed that there were no significant differences between the genotypes distribution within cervical lesions and cancer (p>0.050), neither when adjusting the analysis for age by logistic regression (data not shown).

### ATM G5557A polymorphism

Table III shows the distribution of the *ATM* G5557A genotypes and their frequencies in cervical specimens. From 429 cases, 71.6% were found to be homozygous for G allele, 25.6% were heterozygous, and 2.8% were homozygous for A allele. Statistical analysis revealed significant differences between A carriers versus GG homozygous in women with LSIL (p=0.044; OR=1.94; 95% CI 1.01–3.74). Logistic regression adjusting for age revealed no statistical differences (data not shown).

## Discussion

HPV is the etiological agent of cervical cancer and its precursor lesions and viral genome integration into the host genome is considered to be the crucial step of cervical carcinogenesis (8,9). Beyond the genetic events that can affect genome integrity, viral integration is considered to have a high impact on cell de-regulation and its control is extremely important to prevent the development of neoplastic cells (16,18,45).

Viral DNA integration is though to occur by the erratic activation of DNA repair mechanisms upon the formation of DSBs (14–17). The ATM protein is a crucial kinase for cell cycle checkpoints regulation and activation of cellular responses to DNA damage (21,28). ATM is usually activated through the p53-dependent pathway mainly by the 53BP1 and leads to a complex pathway of different events which will arrest the cell cycle and initiate DNA repair processes (24,27,29,46). When DNA repair processes are inactivated or deficiently activated, cell cycle arrest and DNA repair might not occur, and cells start to accumulate errors promoting genetic instability, which can lead to the development of cancer (18). Recently, polymorphisms on 53BP1 and ATM genes were studied for their role on cancer development, and since oncogenic viruses might interact with these proteins there is a great need to evaluate the possible role of polymorphisms on these proteins in viral associated cancer development.

### 53BP1 C1236G polymorphism

It has been suggested that a 53BP1 genetic polymorphism involving the transition C→G at position 1236, leading to an amino acid shift (D353E) on the BRCT domain of p53bp1 (44), interferes with the ability to interact with the DNA-binding core of p53 (19,22,27). Therefore, p53-dependent DNA repair mechanisms might not be activated efficiently (47). This polymorphism was recently associated with increased risk for breast cancer development in individuals carrying the Pro/Pro genotype of the p53 codon 72 (43). Considering the cervical carcinogenesis model, if the affinity of the p53BP1 for p53 is lost, HPV-infected cells will be more prone to HPV E6-mediated p53 degradation, increasing the risk of cancer development. Our study revealed no significant association between the 53BP1 genotypes and the development of cervical cancer. A crude analysis of OR reveals a tendency for increased risk of LSIL, nevertheless, in our study no statistically significant results were obtained.

Surprisingly, our study revealed significant differences on the p53bp1 D353E genotypes comparing our control population with data from other studies: Frank *et al* reported in a German population that 9.9% were GG homozygous, 42.6% were GC and 47.6% were CC homozygous (44); and Ma *et al* reported 18.2% as GG homozygous, 50.9% GC and 30.9% were CC homozygous in a Chinese population (43). Therefore it is necessary to point out that these results may be influenced by ethnic variables.

### ATM G5557A polymorphism

Previous reports have shown that a G>A transition at position 5557, which produces an amino acid shift (D1853N), is supposed to affect the normal splicing of ATM leading to the expression of a less effective protein (34–36,40). In normal conditions, ATM, one of the major sensors of DNA damage, activates a complex pathway of different events, leading to the cell cycle checkpoint activation and arrest for DNA repair (16). When activation of ATM does not occur efficiently, cell cycle arrest and DNA repair are void and the accumulation of DNA errors, such as, integration of DNA sequences contribute to cellular instability, that can lead to the development of neoplastic cells. Studies revealed that the 5557A is associated with hereditary non-polyposis colorectal cancer in carriers of germline MLH1 and MSH2 mutations (41), and with the development of bilateral breast cancer (36). Our study revealed an increased risk for the ATM GG homozygous to the development of LSIL (OR=1.94; p=0.044). Although, logistic regression adjusted for age revealed no statistical significant differences, our results reveal that this polymorphism should be further studied to clarify its role in the development of cervical cancer.

In conclusion, over the past 10 years, our group has been studying the role of genetic polymorphism in cancer development (48–54). This is the first study to evaluate the role of the ATM D1853N (5557G>A) and p53bp1 D353E (1236C>G) polymorphisms in the development of cervical cancer in Portugal.

Although we did not find significant differences, our data reveal a trend for increased risk of LSIL of both polymorphisms that, if corroborated by a study with higher number of samples, could contribute to clarify if LSIL lesions coud be considered only HPV-infected cells or a true pre-cancer lesion. Hence, our results support the need for further molecular studies to evaluate the role of polymorphism on the DNA damage repair pathways to the predisposition to cervical cancer.

## Figures and Tables

**Table I:** Characteristics of the study population.

Cytological classification	Median age (± SD)
Total (n=429)	45.0±13.7
Normal (n=280)	45.0±13.2
All lesions (n=149)	
LSIL (n=70)	44.0±14.8
HSIL/CIS (n=70)	45.0±13.7
ICC (n=9)	47.0±18.8

**Table II:** Distribution and statistical analysis of p53bp1 D353E (53BP1 1236G>C) genotypes.

	GG, n (%)	GC, n (%)	CC, n (%)	P-value	OR (95% CI)
All Cases					
Normal (n=280)	99 (35.4)	132 (47.1)	49 (17.5)		Reference
All lesions (n=149)	65 (43.6)	63 (42.3)	21 (14.1)	0.093	1.42 (0.94–2.12)
LSIL (n=70)	33 (47.1)	27 (38.6)	10 (14.3)	0.069	1.63 (0.96–2.77)
HSIL/CIS (n=70)	29 (41.4)	32 (45.7)	9 (12.9)	0.346	1.29 (0.76–2.20)
ICC (n=9)	3 (33.3)	4 (44.4)	2 (22.2)	0.602	0.91 (0.22–3.74)

LSIL, low-grade squamous intraepithelial lesion; HSIL, high-grade squamous intraepithelial lesion; ICC, invasive cervical cancer; Chi-square analysis (χ^2^) was performed by comparing C carrier vs. GG homozygous; P, Pearson χ^2^; OR, odds ratio; CI, confidence interval.

**Table III:** Distribution and statistical analysis of ATM D1853N (ATM 5557G>A) genotypes.

	GG, n (%)	GA, n (%)	AA, n (%)	P-value	OR (95% CI)
All Cases					
Normal (n=280)	194 (69.3)	79 (28.2)	7 (2.5)		Reference
All lesion (n=149)	113 (75.8)	31 (20.8)	5 (3.4)	0.152	1.39 (0.89–2.19)
LSIL (n=70)	57 (81.4)	9 (12.9)	4 (5.7)	0.044	1.94 (1.01–3.74)
HSIL/CIS (n=70)	49 (70.0)	20 (28.6)	1 (1.4)	0.908	1.03 (0.59–1.83)
ICC (n=9)	7 (77.8)	2 (22.2)	-	0.449	1.55 (0.32–7.62)

LSIL, low-grade squamous intraepithelial lesion; HSIL, high-grade squamous intraepithelial lesion; ICC, invasive cervical cancer; Chi-square analysis (χ^2^) was performed by comparing A carrier vs. GG homozygous P, Pearson χ^2^; OR, odds ratio; CI, confidence interval.
